# Development of emergent processes and threshold of consciousness with levels of processing

**DOI:** 10.3389/fpsyg.2024.1337589

**Published:** 2024-07-10

**Authors:** Ryoichi Watanabe, Yusuke Moriguchi

**Affiliations:** Graduation School of Letters, Kyoto University, Kyoto, Japan

**Keywords:** children, visual consciousness, backward masking, awareness, level of processing

## Abstract

**Introduction:**

The transition of experience from unconscious to conscious, the emergent process, is a crucial topic in consciousness studies. Three frameworks exist to explain the process: (1) consciousness arises in an all-or-none manner; (2) consciousness arises gradually; (3) consciousness arises either all at once or gradually, depending on the level of stimulus processing (low- vs. high-level). However, the development of emergent processes of consciousness remains unclear. This study examines the development of emergent processes of consciousness based on the level of stimulus processing framework.

**Methods:**

Ninety-nine children (5–12 year-olds) and adults participated in two online discrimination tasks. These tasks involved color discrimination as lower-level processing and number magnitude discrimination as higher-level processing, as well as backward masking with stimulus onset asynchronies (SOAs) varying from 16.7 to 266.7 ms. We measured objective discrimination accuracy and used a 4-scale Perceptual Awareness Scale (PAS) to assess subjective awareness. We fit the data to a four-parameter nonlinear function to estimate the center of the slope (threshold) and the range of the slope (gradualness, the measure of emergent process of consciousness) of the model.

**Results:**

The results showed the threshold of objective discrimination was significantly higher in 5–6 year-olds than in 7–12 year-olds, but not of subjective awareness. The emergent process of objective discrimination in the number task was more gradual than in the color task.

**Discussion:**

The findings suggest that the thresholds of subjective awareness in 5–6 year-olds and objective discrimination in 7–9 year-olds are similar to those in adults. Moreover, the emergent processes of subjective awareness and objective discrimination in 5–6 year-olds are also similar to those in adults. Our results support the level of processing hypothesis but suggest that its effects may differ across developmental stages.

## 1 Introduction

Visual consciousness means subjective and phenomenal visual experience (e.g., what it is like to see an image) (Koch et al., 2016). Transitioning from unconscious to conscious, the emergence of visual consciousness is a prominent topic in consciousness studies (Baars, 2005; Del Cul et al., 2007; Koch and Preuschoff, 2007; Dehaene and Changeux, 2011; Sandberg et al., 2011; Windey et al., 2013; Koch et al., 2016). Research on visual consciousness’s emergent processes and neural mechanisms has focused on studies in human adults and macaques (Koch et al., 2016). However, how they develop is almost unknown. Research on the developmental aspects of consciousness has increased recently, focusing primarily on the origins of consciousness in fetuses and newborns (Bayne et al., 2023). However, research on the developmental changes afterwards is lacking. Many consciousness researchers believe that understanding the developmental aspects of visual consciousness is essential for consciousness theories (Mashour et al., 2020; Seth and Tim, 2022). The present study focused on the developing emergent process and threshold of visual consciousness in 5–12 year-olds and adults.

### 1.1 Review of literature

The emergent processes and thresholds of consciousness are vital to examining the transformation from unconsciousness to consciousness. Masking methods have been used widely in examining the visual consciousness’s emergent process and thresholds (Sandberg et al., 2011; Windey et al., 2013; Thiruvasagam and Srinivasan, 2021). The emergent process and threshold of consciousness have been examined using subjective awareness and objective discrimination of task performance (Sandberg et al., 2011). Subjective awareness is measured by the two choices of awareness or unawareness of the stimulus or by the Perceptual Awareness Scale (PAS), which assesses perceptual awareness in a graded manner (Ramsøy and Overgaard, 2004). By using PAS, we can measure the presence and intensity of awareness in a graded manner. As the duration of the stimulus presentation increases, the subjective awareness rate and intensity increases (Sandberg et al., 2010, 2011; Windey et al., 2013). The task accuracy measures objective discrimination performance and d’ using signal detection theory. The d’ of the signal detection theory is often used as a measure of stimulus discrimination performance; the larger the d’, the greater the discrimination performance (Macmillan and Douglas Creelman, 2004). The signal detection theory involves calculating four indicators: hit, miss, false alarm, and correct rejection, based on the stimulus combinations between presented and responded. The d’ value is calculated from the difference between the *z*-scores (or standard deviations) of the hit rate and the false alarm rate. For example, in the case of a stimulus color judgment task, if a red stimulus is presented and the participant responds that they saw a red stimulus, a hit is indicated; if the participant responds that they saw a blue stimulus, the response is a miss. Conversely, if a blue stimulus is presented and the participant responds that they saw a red one, this indicates a false alarm, and if the participant responds that they saw a blue stimulus, the response is a correct rejection. As stimulus presentation duration increases, the objective discrimination performance increases (Sandberg et al., 2010, 2011; Windey et al., 2013).

#### 1.1.1 Theory of consciousness

There are three leading positions regarding the process of transitioning from unconscious to conscious (Jimenez et al., 2020): (1) consciousness arises all-or-none (Sergent and Dehaene, 2004; Del Cul et al., 2007; Sekar et al., 2013; Asplund et al., 2014), supported by Global Neuronal Workspace Theory (GNWT; Dehaene and Naccache, 2001; Dehaene and Changeux, 2011); (2) consciousness arises gradually (Ramsøy and Overgaard, 2004; Overgaard et al., 2006; Seth et al., 2008; Pretorius et al., 2016), supported by Recurrent Process Theory (RPT; Lamme, 2006); (3) consciousness arises either all at once or gradually depending on the level of stimulus processing (Windey et al., 2013; Anzulewicz et al., 2015; Binder et al., 2017; Derda et al., 2019; Jimenez et al., 2019, 2021), supported by Level of Processing Hypothesis (LoPH; Windey et al., 2013; Windey and Cleeremans, 2015). The next section explains each position and theory in more details. The position suggesting that consciousness emerges as all-or-none contradicts the one stating that consciousness emerges gradually. However, a comprehensive position exists which covers both ideas by implying that consciousness emerges differently depending on the level of stimulus processing.

#### 1.1.2 All-or-none emergent process and GNWT

The all-or-nothing position assumes that the stage of consciousness is binary, either aware or unaware. *Global Neuronal Workspace Theory* (GNWT) supports this position. GNWT postulates that when the intensity of a stimulus exceeds a certain threshold, the stimulus reaches the global workspace and can be consciously accessed (all-aware) (Dehaene and Naccache, 2001; Dehaene and Changeux, 2011).

Many empirical studies support this position (Del Cul et al., 2006, 2007; Sekar et al., 2013; Asplund et al., 2014). Global workspace is a concept similar to working memory; when information accesses the global workspace, it can be consciously used for other modalities such as reports and memory (Baars, 2005). Del Cul et al. (2006) showed that the longer the target-mask stimulus onset asynchrony (SOA), the higher the objective performance and subjective awareness rating. Moreover, they showed that the trajectory of the objective performance and subjective visibility rating were sigmoidal curves centered on the threshold, as well as that the emergent process of visual consciousness follows a sigmoid curve, suggesting that visual consciousness emerges as all-or-none. Thus, GNWT suggests that objective discrimination and subjective awareness are all or none.

The GNWT argues that information is accessed by consciousness when transferred through the frontal–parietal network to the frontal lobes and the whole brain (Dehaene and Naccache, 2001; Dehaene and Changeux, 2011). Thus, it is suggested that the development of the frontal–parietal network and frontal lobes is related to the emergent process of consciousness (e.g., the threshold or the precision).

The volume and density of gray and white matter in the frontal and parietal lobes peaks during childhood (Giedd et al., 1999; Nagy et al., 2004; Tanaka et al., 2012). Moreover, the activity and connectivity of the frontal and parietal lobes become stronger during childhood and adolescence (Adleman et al., 2002; Kwon et al., 2002; Gogtay et al., 2004). Finally, activity in frontal–parietal regions begins to function from early childhood (Moriguchi and Hiraki, 2013), with weak activity in frontal–parietal regions during inhibition tasks, reflected by the coupling of the frontal–parietal network (Mehnert et al., 2013). Considering the development of these frontal–parietal networks, the threshold in the emergent process of consciousness is predicted to become smaller and more precise as the networks develop.

#### 1.1.3 Gradual emergent process and RPT

The gradual position assumes that the stage of consciousness is not binary but gradual from non-aware to all-aware. *Recurrent processing theory* (RPT) supports this position. RPT postulates that as a stimulus is processed progressively, it becomes more explicit to consciousness (Lamme, 2006). In other words, as the intensity of the stimulus increases (e.g., SOA or stimulus contrast), the conscious experience of that stimulus becomes clearer. Many empirical studies support this position (Christensen et al., 2006; Overgaard et al., 2006; Sandberg et al., 2010, 2011). Sandberg et al. (2010) showed that using the subjective measure PAS, intermediate awareness responses, such as slightly visible or mostly visible, increased for SOAs around the threshold. Our study defines the threshold as the central point of the model’s slope. The gradual emergent process is accompanied by its threshold.

Furthermore, they showed that the subjective measures, PAS, confidence rating, and weighting predicted objective discrimination performance. Sandberg et al. (2011) developed a sigmoid function that fits objective discriminant performance and subjective awareness rating. This function can be used to estimate the threshold and steepness of the slope of the model, indicating whether the model is all-or-none or gradual. Subjective awareness rating and objective discrimination performance were showed to increase gradually as SOA increased, which suggests that visual consciousness emerged gradually.

The RPT argues that the recurrent loop of the visual cortex produces a clearer visual consciousness, implying that the development of the visual cortex and the recurrent loop is related to the emergent process of consciousness.

Other studies indicate a recurrent loop in visual information after 7–8 months (Nakashima et al., 2021). The total number and density of synapses peaks at 1 year of age and decreases to the same level as adults at about 10–11 years of age (Huttenlocher et al., 1982; Huttenlocher, 1990). Although there is not necessarily a relationship between synaptic density and cognitive function, based on the recurrent loop structure, the emergent visual consciousness process is predicted to develop from infancy to childhood and remain similar during adulthood.

#### 1.1.4 All-or-none/gradual emergent process and LoPH

Windey et al. (2013) and Windey and Cleeremans (2015) integrated the contradictions between these two positions by varying the stimulus or task’s processing level and presented the level of processing hypothesis (LoPH). According to LoPH, when the task requires higher-order processing of the stimulus, the emergent process of consciousness is either all-or-none, and when the task requires lower-order processing of the stimulus, the emergent process of consciousness is gradual (Windey et al., 2013; Windey and Cleeremans, 2015). The level of processing corresponds to feed-forward brain processing of visual information, with higher processing levels referring to the meaning or category of the visual object and lower processing levels referring to the shape or color of the visual object (Hochstein and Ahissar, 2002; Windey and Cleeremans, 2015). Windey et al. (2013) examined the difference in the slope steepness of objective discrimination performance and subjective awareness rating between color judgments and number magnitude judgments. They used the color judgements as the lower-order processing condition and the number magnitude judgements as higher-order one, with numbers in different colors as stimuli. They showed that the steepness of the slope of the model in the low-order processing condition was more gradual than that in the high-order condition for both objective discrimination performance and subjective awareness rating. Empirical studies that support the all-or-none process used tasks that require judgments about the meaning of stimuli (e.g., the meaning of letters or the magnitude of numbers) (Del Cul et al., 2006, 2007; Sekar et al., 2013; Asplund et al., 2014). In contrast, empirical studies that support a gradual process of consciousness used tasks that require judgments of stimulus characteristics (e.g., the color of letters or the shape of a stimulus) (Christensen et al., 2006; Overgaard et al., 2006; Sandberg et al., 2010, 2011).

Considering the all-or-none, gradual, and LoPH positions, the threshold for visual consciousness is predicted to decrease with age from preschool to school, and similar to adults in late childhood. However, no previous studies examine the developmental differences in the emergent process of consciousness with the level of stimulus processing.

**Figure 1:**
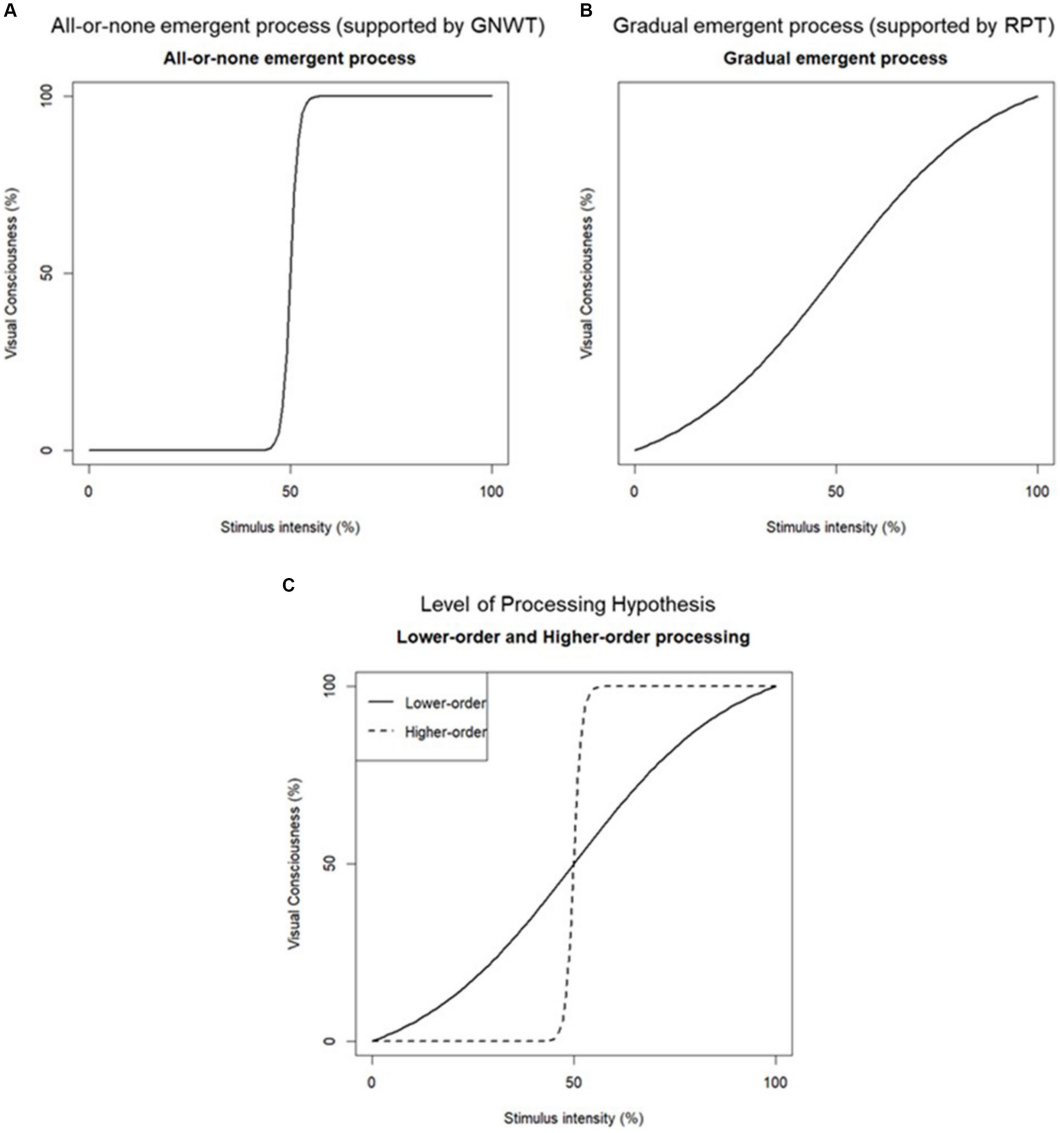
Models of the emergent process of visual consciousness. The model of the all-or-none emergent process **(A)**. The model of the gradual emergent process **(B)**. The model of the level of processing hypothesis, with solid line model representing the lower-order processing model and the dotted one showing the higher-order processing model **(C)**. The thresholds are 50%, the stimulus intensity (%; *x*-axis), and the visual consciousness (%; *y*-axis).

Figure 1A shows the model of the all-or-none emergent process, and Figure 1B shows the gradual emergent process. The level of processing model is drawn in Figure 1A for higher-order processing stimuli and in Figure 1B for lower-order processing stimuli (Figure 1C).

#### 1.1.5 Development of the emergent process and threshold of consciousness

The development of the emergent processes of consciousness is still unclear. Research with children has focused on the objective discrimination performance and thresholds of visual stimuli using the backward masking paradigm. In this paradigm, the target and mask stimuli are presented in order, with manipulated time between the two stimuli (e.g., SOA) (Breitmeyer and Ogmen, 1984). When this time is shorter, for example 20 ms, both objective discrimination and subjective awareness are low. On the other hand, as the time between stimuli increases (e.g., 100 ms), the objective discrimination and subjective awareness of the stimuli also increase (Del Cul et al., 2007; Gelskov and Kouider, 2010; Sandberg et al., 2010, 2011; Kouider et al., 2013; Windey et al., 2013; Anzulewicz et al., 2015; Binder et al., 2017; Jimenez et al., 2019; Thiruvasagam and Srinivasan, 2021). Previous studies have shown that young children have lower objective discrimination performance and larger thresholds for discrimination of letter stimuli than school children and adults (Welsandt et al., 1973; LeBlanc et al., 1992; Macchi et al., 2003). They also showed that the objective threshold of children decreased from 5–16 year-olds and was similar to 22 year-olds, but the performance increased from 5–22 year-olds (Welsandt et al., 1973). Recently, Watanabe and Moriguchi (2023) showed that young children have larger thresholds for objective discrimination and subjective awareness than adults and similar emergent processes of objective discrimination and subjective awareness to adults on a form judgment task (i.e., judgment of the shape of stimuli) categorized in the lower-level processing. Thus, it is consistent with previous studies that objective discrimination performance increases and thresholds decrease from preschool age (Welsandt et al., 1973; LeBlanc et al., 1992; Macchi et al., 2003; Watanabe and Moriguchi, 2023).

However, two issues raised in previous studies should be addressed. The first is that previous research examined only the thresholds and emergent processes of subjective awareness and objective discrimination in lower-order but not higher-order processing stimuli. Watanabe and Moriguchi (2023) showed that the threshold for subjective awareness of form stimuli at ages 5–6 is larger than that of adults, but the emergent process is similar. Although form judgments are categorized as low-order processing, examining low- and high-order processing within the same children is necessary. This study used color judgments as low-order and number magnitude judgments as high-order processing (Windey et al., 2013). For example, young children with immature executive function may have larger thresholds for number magnitude judgments and lower performance than for color judgments compared to other age groups (Prager et al., 2016), and their emergent processes may follow a different trajectory than those of adults. The second issue that should be addressed is the timing when developmental changes occur. Few studies have assessed the developmental changes of emergent processes, and it is unclear whether the emergent processes develop significantly from early childhood to childhood or later. Developmental trajectories may also differ depending on the level of processing of stimuli or tasks (i.e., low- vs. high-level processing).

### 1.2 The present study

#### 1.2.1 Purpose of this study

This study examines the developmental aspect of the level of processing hypothesis in visual consciousness. Thus, we analyzed the thresholds and emergent processes of visual consciousness with the level of stimulus processing and age differences. We focused on children aged 5–12 years and adults. Based on previous research, 5 year-olds can respond to objective discrimination and subjective awareness in backward masking tasks (Welsandt et al., 1973; LeBlanc et al., 1992; Macchi et al., 2003; Watanabe and Moriguchi, 2023). Furthermore, the threshold and performance for objective discrimination have increased significantly between the ages of 5–12 years (Welsandt et al., 1973; LeBlanc et al., 1992; Macchi et al., 2003). We adopted the masking task used by Binder et al. (2017). They used colored numbers as the target stimulus, with the color judgment task as the lower-level processing condition and the number magnitude judgment task as the higher-level processing condition.

First, we measured objective discrimination accuracy and subjective awareness of the stimuli using the four-point PAS scale (Overgaard et al., 2006). Children aged 5–6 can also answer PAS (Watanabe and Moriguchi, 2023). Second, we fit the obtained objective discrimination accuracy and subjective awareness ratings for both conditions with a four-parameter nonlinear psychometric function (Sandberg et al., 2011; Windey et al., 2013; Thiruvasagam and Srinivasan, 2021). Finally, we measured the center of the slope of the fitted psychophysical function as the threshold and the steepness of the slope as the gradualness of the emergent process of consciousness. The steeper the slope indicates an all-or-none dichotomous transition.

Based on the level of processing hypothesis (Windey et al., 2013; Anzulewicz et al., 2015), we predicted that the steepness of the slope for the objective discrimination and subjective awareness of the lower-level color judgment task would be larger than that for the higher-level number magnitude judgment task in 5–12 year-olds and adults (Hypothesis 1). Furthermore, based on the previous research about children’s thresholds of objective discrimination and subjective awareness (Watanabe and Moriguchi, 2023), we predicted that the thresholds for objective discrimination and subjective awareness for the lower-order color and the higher-order number magnitude judgment tasks decrease with age (Hypothesis 2). We examined the interaction between the level of processing of stimuli and age (Explorative Hypotheses 1 and 2).

#### 1.2.2 Hypotheses

*Hypothesis 1*. The gradualness of the slope of the objective discrimination and the subjective awareness for lower-level color judgment tasks is larger than that for higher-level number magnitude judgment tasks in 5–12 year-olds and adults.

*Hypothesis 2*. The thresholds of the objective discrimination and the subjective awareness for lower-level color judgment tasks and higher-level number magnitude judgment tasks decrease with age.

*Explorative Hypothesis 1*. There is an interaction in the gradualness of the slope between the level of processing of stimuli and age or not.

*Explorative Hypothesis 2*. There is an interaction in the threshold between the level of processing of stimuli and age or not.

## 2 Method

### 2.1 Participants

We recruited 126 participants in four age groups (30 5–6 year-olds, 36 7–9 year-olds, 29 10–12 year-olds, and 31 adults). Twenty-seven participants (seven 5–6 year-olds, ten 7–9 year-olds, three 10–12 year-olds, and seven adults) did not complete the entire trial due to computer errors or omission of participant number. Therefore, our final sample consisted of 99 participants in the four age groups (23 5–6 year-olds, 26 7–9 year-olds, 26 10–12 year-olds, and 24 adults (mean age = 48.13, SD = 6.89)) (Table 1). The sample size estimation using the G*Power 3.1.9.7 (Faul et al., 2007) showed that 19 participants per age group were enough (effect size *f* = 0.25, *α* error probability = 0.05, Power = 0.95, number of groups = 4, number of measures = 2, correlation among repeated measures = 0.5, and nonsphericity correction *e* = 1) focusing on the main effect of the task.

**Table 1:** Participant information.

	Total	Computer error	Analyzable data	Catch trials	Model fitting	Final
5–6 year-olds	30	−7	23	−1	−4	18
7–9 year-olds	36	−10	26	0	−2	24
10–12 year-olds	29	−3	26	0	−4	22
Adults	31	−7	24	0	−3	21
All	126	−27	99	−1	−13	85

The Ethics Committee of the Unit for Advanced Studies of the Human Mind, Kyoto University, approved the study procedure (No. 2-P-11). Written informed consent was obtained from the adult participants and the parents of all child participants.

### 2.2 Stimuli and apparatus

We used four different numbers (1, 3, 7, 9) in four different colors (RGB-values of red = 255, 0, 0; light red = 255, 100, 100; blue = 0, 0, 255; light blue = 100, 100, 255) as target stimuli based on previous research (Sandberg et al., 2011; Windey et al., 2013; Binder et al., 2017; Derda et al., 2019; Thiruvasagam and Srinivasan, 2021). We used random multicolored patches generated from four colored rectangles (size 50 × 50 pixels) as a backward mask stimulus based on previous research (Sandberg et al., 2011; Windey et al., 2013; Binder et al., 2017; Derda et al., 2019; Thiruvasagam and Srinivasan, 2021). We presented the stimuli on a grey background (RGB-values of grey = 125, 125, 125). Participants viewed the stimulus from about 60 cm from their personal computers (PCs) with a 13- to 16-inch, 60 Hz refresh rate and an 800 × 600 pixels resolution. We set the size of the target stimulus at the height of 0.3 (about 5 degrees of visual angle) and the size of the mask stimulus at the height of 0.5 (about 8.5 degrees of visual angle) on the screen. We made the tasks using PsychoPy (Psychology Software Tools, Inc., 2002) and Pavlovia^1^.

### 2.3 Procedure

Before the experiment, participants (or their parents) accessed the task URL through a web browser and downloaded the tasks on the Pavlovia. Participants completed two tasks in approximately 50–60 min. For 5–6 year-old participants, we asked the participants to respond verbally or point to the answer in each trial and their parents to click the appropriate display location for the participants. Our pilot experiment demonstrated that 5–6 year-olds had difficulty responding using a computer mouse. Participants in the other age groups responded by themselves with a mouse. The experimenter directly instructed all 5–6 year-olds about the task and observed that the parents and children conducted the tasks via the Zoom meeting app to check that they precisely clicked their children’s answers.

**Figure 2:**
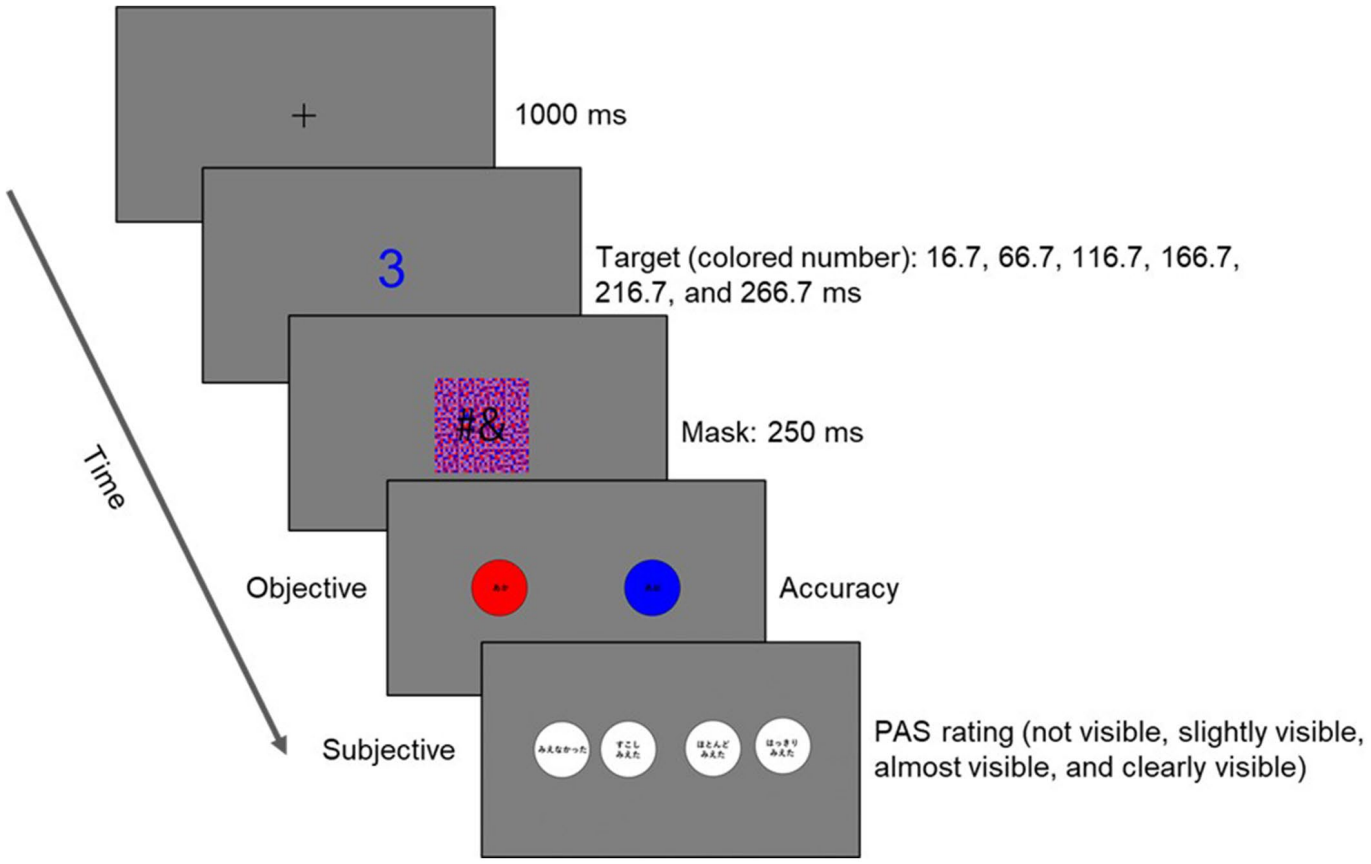
Design of the stimuli and procedure of the task. The target stimuli were colored numbers, and the masking stimuli were random multicolored patches. Participants responded to the objective discrimination question, “Was the number red or blue? Was the number larger or smaller than five?” and the subjective awareness question, “How clearly did you see the number?” as the 4-scale PAS.

The design of the procedure, summarized in Figure 2, was similar to previous studies (Sandberg et al., 2011; Windey et al., 2013; Binder et al., 2017; Derda et al., 2019; Thiruvasagam and Srinivasan, 2021). Participants conducted two discrimination tasks (the color and number magnitude judgment tasks) in separate blocks online. Both tasks will involve identical sequences of stimuli. We counterbalanced the order of the tasks among participants. In the color judgment task, participants judged whether the color of the number was red or blue. In the magnitude judgment task, participants judged whether the number was smaller or larger than five. In both tasks, each trial began with a black fixation cross displayed centrally for 1,000 ms. Then, the target stimulus appeared for one of six durations (16.7, 66.7, 116.7, 166.7, 216.7, and 266.7 ms). Then, participants judged whether the number was red/blue or smaller/larger than five by clicking one of the two panels (red and blue or smaller and larger). Then, they evaluated the 4-scale PAS for the target stimulus by clicking one of the four panels (no experience, slight experience, almost experience, and clear experience). Participants conducted 24 trials and one catch trial every four blocks. In the catch trial, the catch instruction, which instructed that participants click one of the four panels (e.g., click “smaller” and “slight experience”), appeared for 2000 ms. Thus, participants conducted 96 and four catch trials in each task.

### 2.4 Data analysis

#### 2.4.1 Data exclusion

We excluded the participants’ data whose performance in the catch trials was lower than 50% (2/4), and the model fitting decision coefficient *R*^2^ was lower than 0.5 after the analysis. The exclusion criterion for catch trials was set at the chance probability of 50%, indicating that the excluded participants were not performing above chance. The exclusion criterion for model fitting was set at 0.5 for *R*^2^ (ranging from 0 to 1.0), representing a midpoint value to ensure that the model fits the data.

#### 2.4.2 Nonlinear models

We fit a four-parameter nonlinear (Eq. 1) to the objective discrimination accuracy and subjective awareness rating (Sandberg et al., 2011).


(1) $$f\left(x\right)=a+(b-a)/(1+\exp.((c-x)/d))$$

Parameters *a* and *b* reflect the lower and upper boundaries of the psychometric function (i.e., *a* = 0 and *b* = 1 in the objective discrimination accuracy; *a* = 1 and *b* = 4 in the subjective awareness rating. Parameters *c* and *d* reflect the threshold and the steepness of the model slope, respectively. Larger parameter *d* indicates a more gradual model slope.

#### 2.4.3 Statistical analysis

First, we analyzed task, age, and SOA differences in objective discrimination performance and subjective awareness rating. We conducted a three-way ANOVA on discrimination performance “sdt d’” of the signal detection theory as objective discrimination performance and PAS in each SOA. The independent variables were tasks (the lower-level color judgment task and the higher-level number magnitude judgment task) and age (5–6 year-olds, 7–9 year-olds, 10–12 year-olds, and adults), and SOA (16.7, 66.7, 116.7, 166.7, 216.7, and 266.7 ms). If there was a significant difference in the interaction, we conducted a *post hoc* analysis.

##### 2.4.3.1 Hypotheses 1 and explorative hypotheses 1

We conducted a two-way ANOVA on the gradualness of the slope of the objective discrimination and subjective awareness. The independent variables were tasks (the lower-level color judgment task and the higher-level number magnitude judgment task) and age (5–6 year-olds, 7–9 year-olds, 10–12 year-olds, and adults). If there was a significant difference in the interaction, we conducted a *post hoc* analysis.

##### 2.4.3.2 Hypotheses 2 and explorative hypotheses 2

We conducted a two-way ANOVA on the threshold of objective discrimination and subjective awareness. The independent variables were tasks (the lower-level color judgment task and the higher-level number magnitude judgment task) and age (5–6 year-olds, 7–9 year-olds, 10–12 year-olds, and adults). If there was a significant difference in the interaction, we conducted a *post hoc* analysis.

## 3 Results

### 3.1 Data exclusion

The final sample for analysis included 99 participants in four age groups (23 5–6 year-olds, 26 7–9 year-olds, 26 10–12 year-olds, and 24 adults (mean age = 48.13, SD = 6.89)).

We excluded one 5–6 year-old child because their catch-trial accuracy was lower than 50% (2/4 trials). We also excluded 13 participants (four 5–6 year-olds, two 7–9 year-olds, four 10–12 year-olds, and three adults) because the model fitting decision coefficient *R*^2^ was lower than 0.5. Then, the data of 85 participants in four age groups (18 5–6 year-olds, 24 7–9 year-olds, 22 10–12 year-olds, and 21 adults) were analyzed for the hypotheses (Table 1). The number of 5–6 year-olds did not reach the initial goal of 19 but was instead held at 18 to meet the recruitment budget limit maintain consistency in recruitment methods.

### 3.2 Subjective awareness and objective discrimination

#### 3.2.1 Perceptual awareness scale

**Figure 3:**
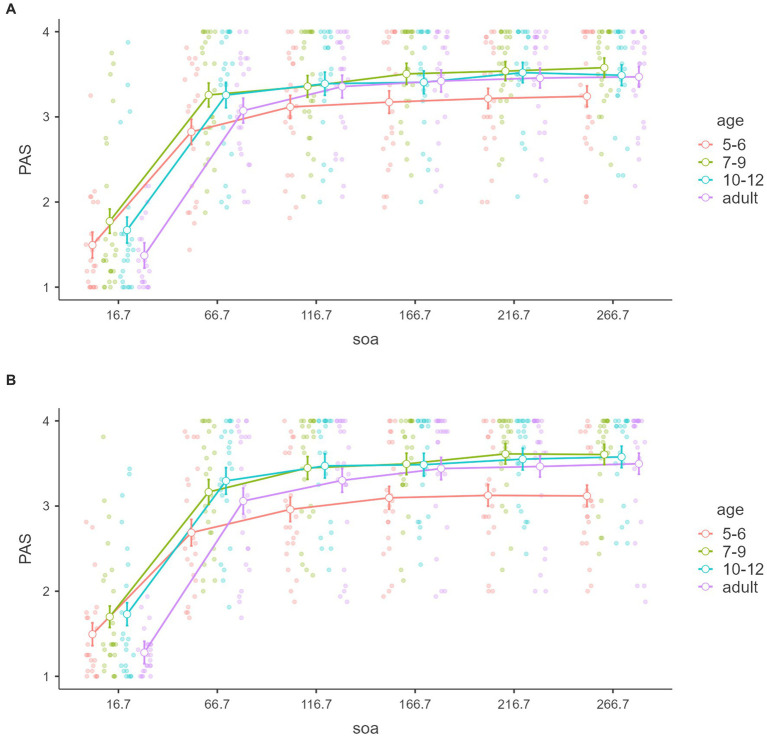
Perceptual awareness scale with tasks. Mean “PAS” of the signal detection theory (*y*-axis) of subjective awareness with SOAs (*x*-axis) and age groups (color groups) of the color and number tasks **(A,B)**. Error bars represent standard error.

**Figure 4:**
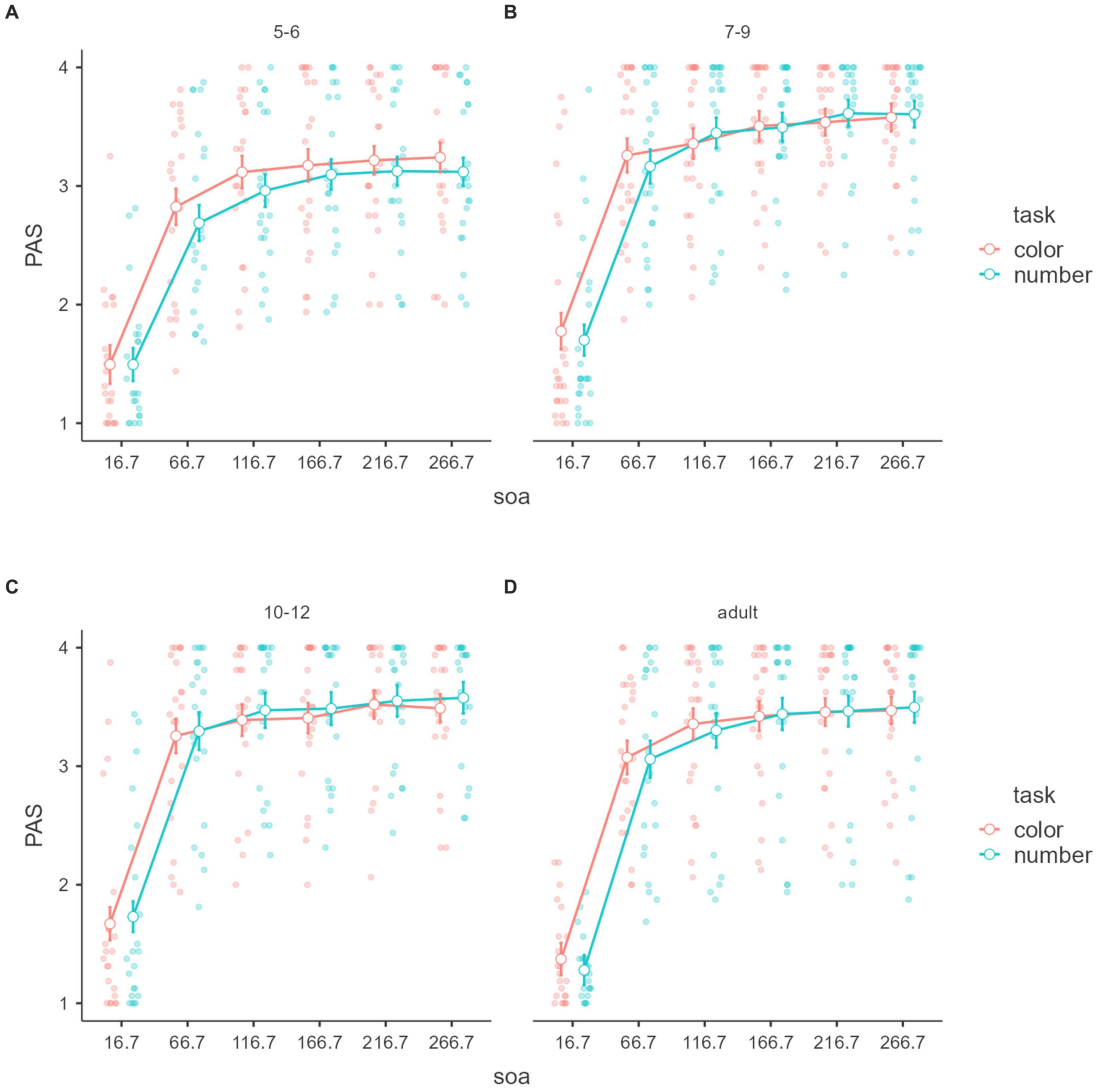
Perceptual awareness scale with age groups. Mean “PAS” of the signal detection theory (*y*-axis) of subjective awareness with SOAs (*x*-axis) and tasks (color groups) of the age groups **(A–D)**. Error bars represent standard error.

Figures 3, 4 show PAS within tasks, SOAs, and between-age groups in subjective awareness. A significant main effect was found for SOA, *F*_(5,440)_ = 466.2214, *p* < 0.001, *η*^2^ = 0.516, partial *η*^2^ = 0.841.

Tukey’s *post hoc* for the main SOA effect analysis showed that the 16.7 ms was significantly lower than the other SOA (*p*s < 0.001), 66.7 ms was significantly lower than the more SOA (*p*s < 0.001), 116.7 ms was significantly lower than 166.7 ms (*p* = 0.001), 216.7, and 266.7 (*p*s < 0.001), 166.7 ms was significantly lower than 216.7 ms (*p* = 0.002) and 266.7 (*p* = 0.001). There was no significant difference between 216.7 and 266.7 (*p* = 0.964).

#### 3.2.2 Signal detection theory d’

**Figure 5:**
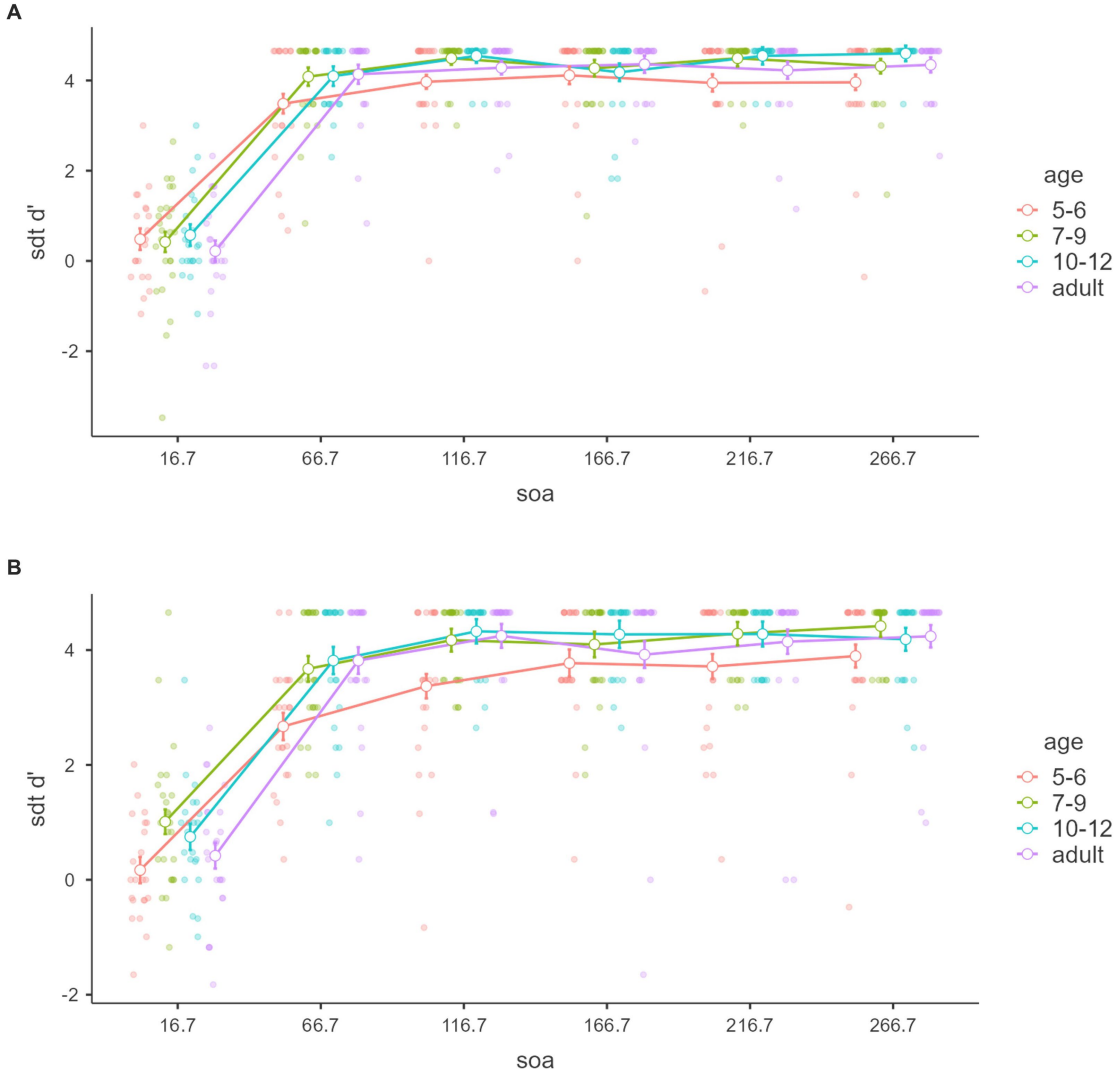
Discrimination ability sdt d’ with tasks. Mean “sdt d’” of the signal detection theory (*y*-axis) of objective discrimination with SOAs (*x*-axis) and age groups (color groups) of the color and number tasks **(A,B)**. Error bars represent standard error.

**Figure 6:**
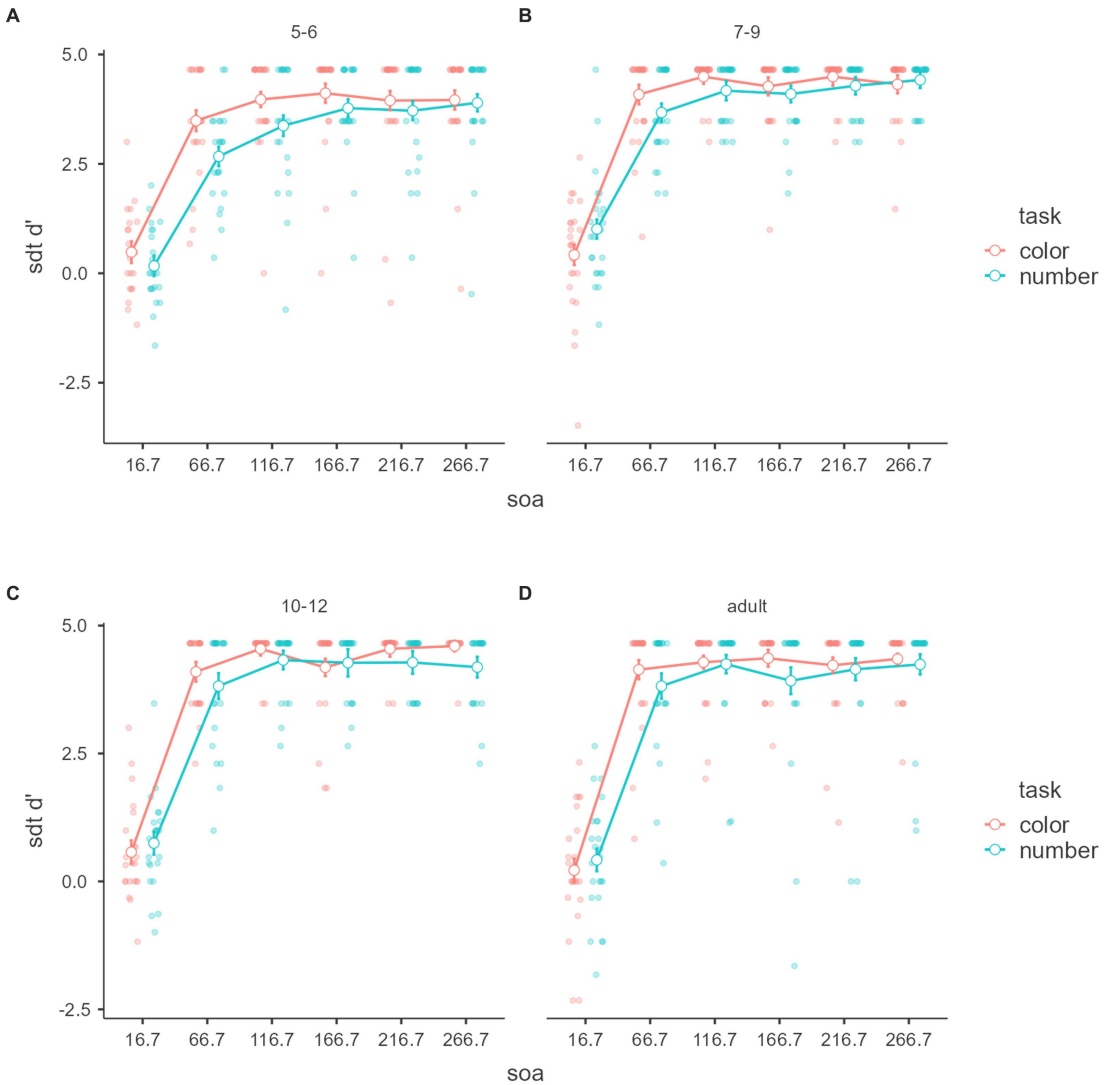
Discrimination ability sdt d’ with age groups. Mean “sdt d’” of the signal detection theory (*y*-axis) of objective discrimination with SOAs (*x*-axis) and tasks (color groups) of the age groups **(A–D)**. Error bars represent standard error.

Figures 5, 6 show signal detection theory d’ within tasks and SOAs and between the age groups in objective discrimination. Significant main effects of task, SOA, and age were found: *F*_(1,88)_ = 4.218, *p* = 0.043, *η*^2^ = 0.003, partial *η*^2^ = 0.046, *F*_(5,440)_ = 703.018, *p* < 0.001, *η*^2^ = 0.641, partial *η*^2^ = 0.889, *F*_(3,88)_ = 4.19, *p* = 0.008, *η*^2^ = 0.016, partial *η*^2^ = 0.125. Moreover, an interaction was detected between task and SOA, *F*_(5,440)_ = 4.054, *p* = 0.001, *η*^2^ = 0.003, partial *η*^2^ = 0.044. The other effect was not significant, *p*s > 0.05.

Tukey’s *post hoc* for the main effect of task analysis showed that d’ in the color task was significantly larger than in the number task (*p* = 0.043). Tukey’s *post hoc* for the main SOA effect analysis showed that the 16.7 ms was significantly lower than the other SOA (*p*s < 0.001), and 66.7 ms was significantly lower than the more SOA (*p*s < 0.001). The other difference was not significant. Tukey’s *post hoc* for the main age effect analysis showed that 5–6 year-olds were significantly lower than 7–9 year-olds and 10–12 year-olds (*p* = 0.017 and *p* = 0.012), not adults (*p* = 0.106). The other age difference was not significant (*p*s > 0.05).

Tukey’s *post hoc* for task and SOA interaction analysis showed that the color 16.7 ms was significantly smaller than the other condition (*p*s < 0.001), not the number 16.7 ms. The color 66.7 ms was significantly smaller than the colors 116.7 (*p* = 0.001), 216.7 (*p* = 0.002), and 266.7 ms (*p* = 0.005) and larger than the number 16.7 (*p* < 0.001) and 66.7 ms (*p* = 0.047). The colors 116.7, 166.7, 216.7, and 266.7 ms were significantly larger than the numbers 16.7 and 66.7 ms (*p*s < 0.001). The number 16.7 ms was significantly smaller than 66.7, 116.7, 166.7ms, 216.7, and 266.7 ms (*p*s < 0.001). The number 66.7 ms was significantly smaller than the number 116.7 (*p* = 0.002), 166.7, 216.7, and 266.7 ms (*p*s < 0.001). The other difference was not significant (*p*s > 0.05).

### 3.3 Hypothesis 1

#### 3.3.1 Parameter *d* (gradualness of the slope)

##### 3.3.1.1 Subjective awareness

**Figure 7:**
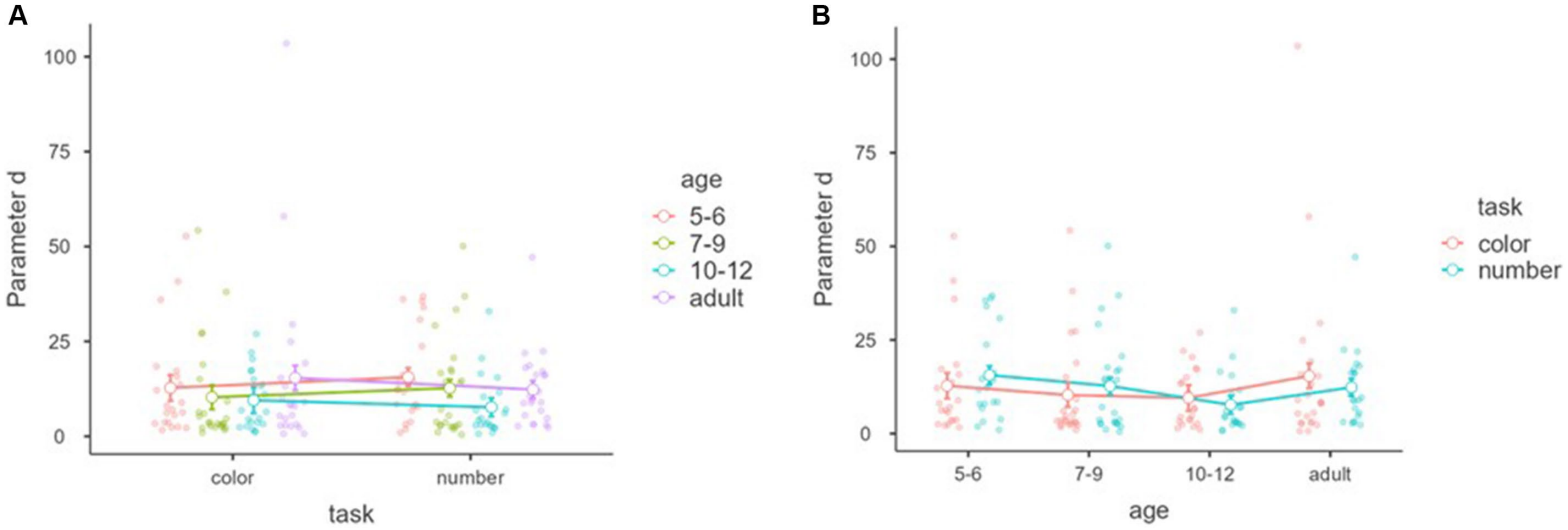
Parameter *d* (gradualness of the slope) with tasks and age groups of the subjective awareness. Mean parameters “d (gradualness of the slope)” of the nonlinear model (*y*-axis) of subjective awareness with tasks (*x*-axis) and age groups (color groups) **(A)**. Mean parameter of “d (gradualness of the slope)” of the nonlinear model (*y*-axis) of subjective awareness with age groups (*x*-axis) and tasks (color groups) **(B)**. Error bars represent standard error.

Figure 7 shows parameter *d* within tasks and between the age groups in the subjective awareness. No significant main effect was found for task and age, and interaction between task and age, *F*_(1,85)_ = 0.00396, *p* = 0.950, *η*^2^ = 0.000, partial *η*^2^ = 0.000, *F*_(3,85)_ = 1.01, *p* = 0.394, *η*^2^ = 0.026, partial *η*^2^ = 0.034, *F*_(3,85)_ = 1.16685, *p* = 0.327, *η*^2^ = 0.009, partial *η*^2^ = 0.040.

##### 3.3.1.2 Objective discrimination

**Figure 8:**
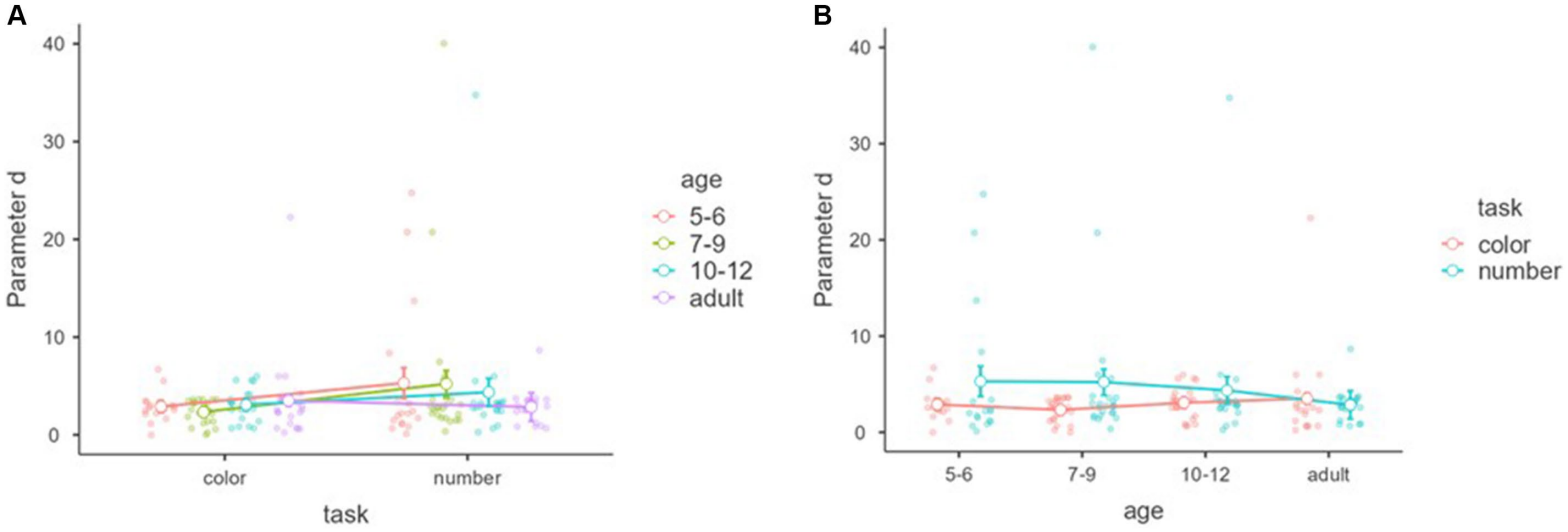
Parameter *d* (gradualness of the slope) with tasks and age groups of the objective discrimination. Mean parameters “d (gradualness of the slope)” of the nonlinear model (*y*-axis) of objective discrimination with tasks (*x*-axis) and age groups (color groups) **(A)**. Mean parameter of “d (gradualness of the slope)” of the nonlinear model (*y*-axis) of objective discrimination with age groups (*x*-axis) and tasks (color groups) **(B)**. Error bars represent standard error.

Figure 8 shows parameter *d* within tasks and between the age groups in the objective discrimination. A significant main effect of task was found, showing that the parameter *d* of the number task was significantly larger than that of the color task *F*_(1,81)_ = 4.01 *p* = 0.049, *η*^2^ = 0.021, partial *η*^2^ = 0.047, although no main effect of age and interaction between task and age, *F*_(3,81)_ = 0.208, *p* = 0.891, *η*^2^ = 0.004, partial *η*^2^ = 0.008, *F*_(3,81)_ = 1.16, *p* = 0.332, *η*^2^ = 0.018, partial *η*^2^ = 0.041.

### 3.4 Hypothesis 2

#### 3.4.1 Parameter *c* (threshold)

##### 3.4.1.1 Subjective awareness

**Figure 9:**
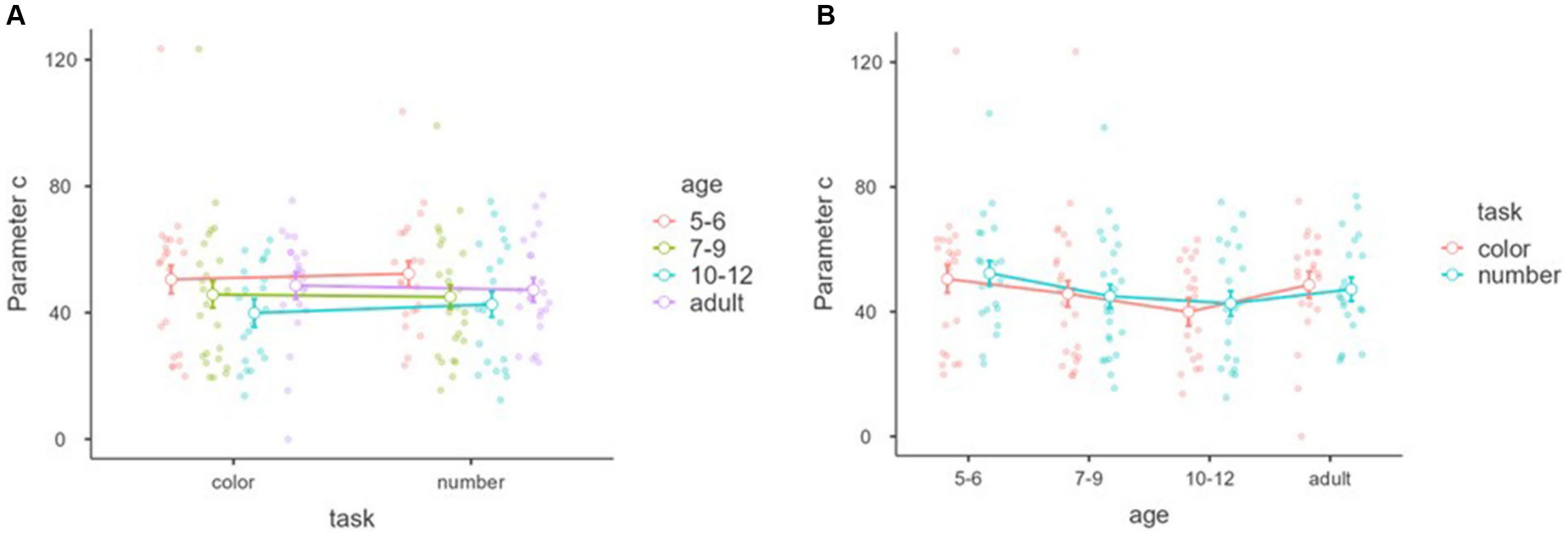
Parameter *c* (threshold) with tasks and age groups of the subjective awareness. Mean parameters “c (threshold)” of the nonlinear model (*y*-axis) of subjective awareness with tasks (*x*-axis) and age groups (color groups) **(A)**. Mean parameter of “c (threshold)” of the nonlinear model (*y*-axis) of subjective awareness with age groups (*x*-axis) and tasks (color groups) **(B)**. Error bars represent standard error.

Figure 9 shows parameter *c* within tasks and between the age groups in the subjective awareness. There was no significant main effect of age and task, and interaction between task and age, *F*_(1,85)_ = 0.0608, *p* = 0.806, *η*^2^ = 0.000, partial *η*^2^ = 0.001, *F*_(3,85)_ = 1.56, *p* = 0.205, *η*^2^ = 0.035, partial *η*^2^ = 0.052, *F*_(3,85)_ = 0.1689, *p* = 0.917, *η*^2^ = 0.002, partial *η*^2^ = 0.006.

##### 3.4.1.2 Objective discrimination

**Figure 10:**
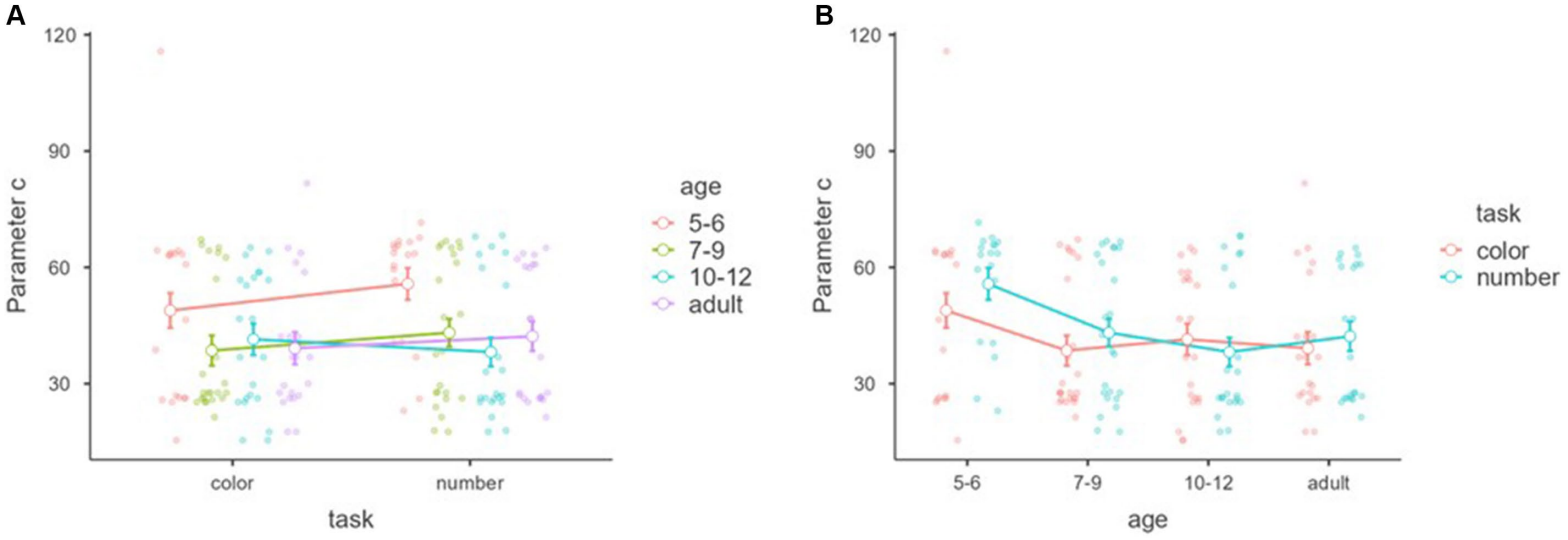
Parameter *c* (threshold) with tasks and age groups of the objective discrimination. Mean parameters “c (threshold)” of the nonlinear model (*y*-axis) of objective discrimination with tasks (*x*-axis) and age groups (color groups) **(A)**. Mean parameter of “c (threshold)” of the nonlinear model (*y*-axis) of objective discrimination with age groups (*x*-axis) and tasks (color groups) **(B)**. Error bars represent standard error.

Figure 10 shows parameter *c* within tasks and between the age groups in the objective discrimination. A significant main effect of age was found, *F*_(3,81)_ = 3.50, *p* = 0.019, *η*^2^ = 0.068, partial *η*^2^ = 0.115, although no main effect of task and interaction between task and age, *F*_(1,81)_ = 1.172, *p* = 0.282, *η*^2^ = 0.006, partial *η*^2^ = 0.014, *F*_(3,81)_ = 0.696, *p* = 0.557, *η*^2^ = 0.010, partial *η*^2^ = 0.025.

Tukey’s *post hoc* analysis showed that the parameter *c* of 5–6 year-olds was significantly larger than that of 7–9 year-olds (*p* = 0.046) and 10–12 year-olds (*p* = 0.028), but not adults (*p* = 0.051). There was no age difference among 7–9 year-olds, 10–12 year-olds, and adults, *p*s > 0.05 (Figure 10).

## 4 Discussion

This study examined how thresholds and emergent processes of objective discrimination and subjective awareness develop with different stimulus processing levels (high-order number task and lower-order color task) using the backward masking task based on the level of processing hypothesis. We set two hypotheses and two exploratory hypotheses. Hypothesis 1 and Explorative Hypothesis 1 examined the development of the emergent process of visual consciousness. Hypothesis 1 was that the gradualness of the slope of the objective discrimination and the subjective awareness for lower-level color judgment task is larger than that for higher-level number magnitude judgment task in 5–12 year-olds and adults. Hypothesis 2 and Explorative Hypothesis 2 examined the development of the threshold of visual consciousness. Hypothesis 2 was that the thresholds of objective discrimination and subjective awareness for lower-level color and higher-level number magnitude judgment tasks decreased with age.

Hypothesis 1 was not supported. No significant main effect of task and interaction between task and age in the gradualness of the slope of the subjective awareness was detected. Moreover, a significant main effect of task was found, although no interaction was detected between task and age in the gradualness of the slope of the objective discrimination. The slope of the number task was significantly more gradual than the color task’s. The results suggest that there are no task or age differences in the emergent process of subjective awareness but task differences in the emergent process of objective discrimination, depending on the level of processing. Furthermore, the results suggest that number magnitude judgments, which was the higher-order processing, occur more gradually than color judgments, which was the lower-order processing.

The results did not support the level of processing hypothesis and were inconsistent with Windey et al. (2013). Contrary to our results, Windey et al. (2013) showed that the slope of the subjective awareness and objective discrimination in the lower-order color judgment task was more gradual than in the higher-order number magnitude judgment task. However, although there was no significant interaction between age and task differences, only our adult results may be consistent with Windey et al. (2013). The mean slope gradualness of subjective awareness and objective discrimination was larger for the color task than for the number task in only adults.

The differences in SOA and the age of the participants may explain why the results of the present study did not replicate the results of the previous studies. First, the SOA in the present study was set larger than in previous studies to allow 5–6 year-olds to perform the task. In the previous study, SOAs were set at 10 ms intervals at less than 100 ms (Windey et al., 2013). The larger SOAs and SOA intervals may have resulted in lower task difficulty and less difference between tasks. Second, the results for adults showed a similar trend to Windey et al. (2013), but the results for children showed the opposite pattern. The inclusion of children can lead to different results between the previous study and the present study.

Hypothesis 2 was partially supported. No significant main effect of age and task or interaction between task and age in the threshold of subjective awareness was found. Moreover, a significant main effect of age was detected, although no main effect of task and interaction between task and age in the objective discrimination was found. The threshold of 5–6 year-olds was significantly larger than that of 7–9 year-olds and 10–12 year-olds, and the other age difference and interaction between task and age were not. The results suggest that the thresholds of objective discrimination become smaller between ages 5–6 and 7–9, but there are no age differences after that. Surprisingly, there were no significant differences between the 5–6 years-old group and adults, which may be explained through the older age of the adult participants in the study. Compared to the face-to-face experiment, the adults taking part online tended to be older. Therefore, it is possible that the performance of the adult participants was lower than in previous studies. The thresholds of subjective awareness and objective discrimination were inconsistent with Watanabe and Moriguchi (2023), which showed that the threshold of objective discrimination and subjective awareness in young children (5–6 year-olds) was larger than that in adults in figure stimuli.

Moreover, the lack of difference between tasks was consistent with the findings of Windey et al. (2013), which showed no task difference in the threshold. The threshold of subjective awareness is developed at age five and is equivalent to that of adults. In contrast, the threshold of objective discrimination is developed at age nine and is equivalent to that of adults.

A possible reason for the discrepancy between subjective awareness and objective discrimination is that their neural bases may differ. The frontal lobes and frontal–parietal network may be related to objective discrimination more than to subjective awareness. Young children’s (aged 5–6 years old) frontal lobes and frontal–parietal networks are more immature than those of adults but the posterior perceptual area are similar to adults (Huttenlocher, 1990; Adleman et al., 2002; Nagy et al., 2004; Tanaka et al., 2012; Mehnert et al., 2013; Moriguchi and Hiraki, 2013), which may result in developmental difference between objective discrimination and subjective awareness. However, subjective awareness is also based on the frontal–parietal network and the frontal lobes (Hatamimajoumerd et al., 2022), with several previous studies showing the relationship of objective discrimination and subjective awareness to the frontal lobes and the frontal–parietal network (Dehaene et al., 2003; Pins and Ffytche, 2003; Lamme, 2006; Del Cul et al., 2007, 2009; Dehaene and Changeux, 2011; van Vugt et al., 2018). Therefore, further research is needed to clarify the differences in the neural basis of objective discrimination and subjective awareness.

Our findings and previous research suggest that level of processing and age have little or no influence on the emergent process of subjective awareness and objective discrimination. That there were no age differences in the emergent process of subjective awareness and objective discrimination in the color and number magnitude tasks was congruent with Watanabe and Moriguchi (2023), which showed no age differences between young children and adults in the figure stimuli, which was the lower-order processing. These findings suggest that the emergent processes of visual consciousness are similar to adults by age 5, regardless of the level of processing.

We examined discrimination ability and response bias. Significant main tasks, SOAs, age differences, and the interaction between tasks and SOAs were found. The *post hoc* analysis of SOA showed a difference between 16.7 ms and 66.7 ms or more and that 66.7 ms did not differ from 116.7 ms or more. The result suggests a threshold between 16.7 ms and 66.7 ms. The *post hoc* analysis of age groups showed that 5–6 year-olds were lower than 7–9 year-olds and 10–12 year-olds, not adults. The result suggests that discrimination performance increases between ages 5 and 9 and maintains between ages 9–12 and adults. These results were consistent with the results of Hypothesis 2.

We showed two new findings. First, no age differences were found in the emergent processes of objective discrimination and subjective awareness. Second, the thresholds of objective discrimination for color and number stimuli decrease developmentally from 5–6 year-olds to 7–12 year-olds. The present results contribute to the knowledge of the development of visual consciousness, which was largely lacking; 5–6 year-olds, like older children and adults, experience subjective awareness, but their objective discrimination of stimuli is undeveloped. Thus, the results suggest that the difference between subjective awareness and objective discrimination decreases with age.

Furthermore, our study demonstrated that the level of processing hypothesis may be consistent for adults, but not for children. Previous studies study, the have indicated a more gradual model for the lower-order processing condition for color judgments compared to the model for the higher-order condition for number magnitude judgments. However, our results showed the opposite for children. These findings can be interpreted in two ways. The first interpretation is that the level of processing hypothesis holds, but its effects may differ across developmental stages. Watanabe and Moriguchi (2023) examined the objective discrimination and subjective awareness of form stimuli in young children and adults by conducting a face-to-face experiment. Their results showed that for both objective discrimination and subjective awareness, the thresholds in young children were higher than those in adults. Thus, children’s responses may be easily influenced by stimuli and experimental methods. The second interpretation is that the level of processing hypothesis itself is suspect. However, we choose to support the first interpretation due to the accumulation of studies that confirm the level of processing hypothesis (Windey et al., 2013; Anzulewicz et al., 2015; Windey and Cleeremans, 2015; Binder et al., 2017; Derda et al., 2019; Jimenez et al., 2021; Thiruvasagam and Srinivasan, 2021). We, however, suggest that the level of processing hypothesis needs to be reconsidered, including its developmental aspects.

This study has some limitations. First, participants conducted the color and number tasks as an online experiment. Differences in the experimental environment may have caused inconsistencies between our results and previous research. In this study, we controlled the experimental environment (e.g., bright and quiet room) and computer setting (e.g., 13–16 inch, 60 Hz, 800*600) as much as possible to reduce between-participants differences. Moreover, in the experiment with 5–6 year-olds, we connected Zoom with the participants to confirm that the parents accurately answered the children’s responses.

Second, a difference in task difficulty may have existed between the color and number tasks. The stimuli and SOA were set the same for the color and number tasks, and the questions and answers were changed. However, the performance of the color task was greater than that of the number task. This difference in performance may have made it easier to make the slope of the number task more gradual than the color task. In our tasks, we increased the SOAs and the intervals of the SOAs of the tasks compared to previous studies to allow children to respond. Although there was no significant interaction between age and task or age and SOA, the results may have reflected differences in performance on the color and number tasks in children. Future studies should further examine the effects of SOA and age in greater detail.

Third, participants responded to how clearly they could see the numbers in the color and number tasks as a subjective awareness. The response criteria may differ between and within participants. For example, participants may have responded with subjective awareness of the “color” of the number, the “size” of the number, and the number “itself.” In the future, direct questions should be asked about the color of the numbers and size, and differences depending on the question should be examined. The PAS is a scale for rating perceptual awareness (e.g., the color of a number) but not for rating cognitive discrimination (e.g., the size of a number). Thus, there may be limitations in directly comparing the two. To examine the level of processing hypothesis, separating lower-order and higher-order processing in perception and lower-order and higher-order processing in cognition may be necessary.

Finally, we considered only the developmental aspects of the level of processing hypothesis, without direct comparisons between the all-or-none position and the gradual position of the emergent consciousness process. One reason is that the parameter d’, which we used as a measure of this process, was a relatively comparative measure of higher-order and lower-order processing conditions. It is necessary to determine a value for parameter d’ that would support an all-or-none emergent process and a gradual emergent process, respectively.

## 5 Conclusion

Despite these limitations, this study is the first to examine the development of emergent processes and the threshold of visual consciousness in childhood with the level of processing. We examined the developmental aspects of the level of processing hypothesis, the most recent theory on the emergent process of visual consciousness and thresholds. The results showed that in objective discrimination, thresholds were higher in 5-6 year-olds than in older children in both the higher- and lower-order processing tasks, but regarding subjective awareness, no age differences were shown between the two tasks. Moreover, in objective discrimination, the emergent process in the higher-order task was more gradual than that in the lower-order task, but there was no task difference in subjective awareness. In conclusion, our study supports the level of processing hypothesis, but notes that its effects may differ across developmental stages. This paper contributes new knowledge to developmental and consciousness research by revealing the development of visual consciousness.

## Data availability statement

The datasets presented in this study can be found in online repositories (https://osf.io/cqezv/).

## Ethics statement

The studies involving humans were approved by the Ethics Committee of the Unit for Advanced Studies of the Human Mind, Kyoto University (No. 2-P-11). The studies were conducted in accordance with the local legislation and institutional requirements. Written informed consent for participation in this study was provided by the participants’ legal guardians/next of kin.

## Author contributions

RW: Conceptualization, Data curation, Formal analysis, Funding acquisition, Investigation, Methodology, Project administration, Resources, Software, Validation, Visualization, Writing – original draft, Writing – review & editing. YM: Conceptualization, Funding acquisition, Methodology, Project administration, Supervision, Validation, Writing – original draft, Writing – review & editing.

## Footnotes

^1^ https://pavlovia.org/
